# SARS-CoV-2 re-infection: development of an epidemiological definition from India

**DOI:** 10.1017/S0950268821000662

**Published:** 2021-03-26

**Authors:** Aparna Mukherjee, Tanu Anand, Anup Agarwal, Harpreet Singh, Pranab Chatterjee, Jitendra Narayan, Salaj Rana, Nivedita Gupta, Balram Bhargava, Samiran Panda

**Affiliations:** 1 Division of Epidemiology & Communicable Diseases, ICMR Headquarters, Delhi, India; 2 Division of Information, Systems and Research Management, ICMR Headquarters, Delhi, India; 3 Department of International Health, Johns Hopkins Bloomberg School of Public Health, Johns Hopkins University, Baltimore, USA; 4 ICMR Headquarters, Delhi, India

**Keywords:** COVID-19, re-infection, viable virus, viral shedding

## Abstract

The current investigation was conducted with the objective to develop an epidemiological case definition of possible severe acute respiratory syndrome-coronavirus-2 (SARS-CoV-2) re-infection and assess its magnitude in India. The epidemiological case definition for SARS-CoV-2 re-infection was developed from literature review of data on viral kinetics. For achieving second objective, the individuals who satisfied the developed case definition for SARS-CoV-2 re-infection were contacted telephonically. Taking available evidence into consideration, re-infection with SARS-CoV-2 in our study was defined as any individual who tested positive for SARS-CoV-2 on two separate occasions by either molecular tests or rapid antigen test at an interval of at least 102 days with one negative molecular test in between. In this archive based, telephonic survey, 58 out of 1300 individuals (4.5%) fulfilled the above-mentioned definition; 38 individuals could be contacted with healthcare workers (HCWs) accounting for 31.6% of the cases. A large proportion of participants was asymptomatic and had higher Ct value during the first episode. While SARS-CoV-2 re-infection is still a rare phenomenon, there is a need for epidemiological definition of re-infection for establishing surveillance systems and this study contributes to such a goal.

Severe acute respiratory syndrome-coronavirus-2 (SARS-CoV-2) re-infection is an emerging concern and there is a need to define it. Therefore, working epidemiological case definition for re-infection was developed and its magnitude was explored via archive-based, telephonic survey. Re-infection with SARS-CoV-2 was defined as two positive tests at an interval of at least 102 days with one interim negative test. Thirty-eight of the 58 eligible patients could be contacted with 12 (31.6%) being HCWs. Majority of the participants were asymptomatic and had higher Ct value during their first episode. To conclude, a working epidemiological case definition of SARS-CoV-2 re-infection is important to strengthen surveillance. The present investigation contributes to this goal and records reinfection in 4.5% of SARS-CoV-2 infected individuals in India.

## Background

SARS-CoV-2 is a novel virus, and the natural history of the disease it causes, coronavirus disease 2019 (COVID-19), is still poorly understood. Multiple reports of possible re-infection have been described in the last few months since the first confirmed report from Hong Kong was published [1]. Since then, there have been reports from India, the USA, Belgium, Ecuador, Qatar and France [2–9]. While these reports are sporadic and case counts are small, they point towards a new phenomenon, which has important public health implications. It remains uncertain as to whether the repeat reverse transcriptase-polymerase chain reaction (RT-PCR) positivity is merely picking up non-viable virus or if it represents a true re-infection or there is a recrudescence of the primary infection [6].

Centers for Disease Control and Prevention (CDC) has considered the duration of 90 days between two positive SARS-CoV-2 RNA along with genomic evidence of re-infection as an investigative criterion to understand the phenomenon of re-infection [10]. The basis of choosing 90 days are the studies showing prolonged viral shedding up to 82 days. However, it is not based on any prospective study documenting re-infection. According to European CDC, re-infection is defined as ‘laboratory confirmation of two infections by two different strains (minimum distance to be determined or supported by phylogenetic and epidemiological data) with timely separated illness/infection episodes’ [11]. While phylogenetic data require molecular testing, epidemiological data include one interim negative test and symptom-free period. The duration of intervening period is not decided.

Keeping in mind that obtaining genomic evidence is resource intensive and not always feasible, our objective was to develop a comprehensive, working epidemiological case definition of SARS-CoV-2 re-infection. Second, to understand the magnitude of re-infection in India, we conducted a retrospective study to explore the suspected SARS-CoV-2 re-infections by applying this case definition.

## Methods

This current study was an archive-based, telephonic survey. Laboratory testing database for COVID-19 available with the Indian Council of Medical Research (ICMR), was used to identify participants eligible for the study. The epidemiological case definition for SARS-CoV-2 re-infection was developed from literature review on SARS-CoV-2 reinfection and data on viral kinetics. Cohort studies reporting viral RNA RT-PCR at multiple time points were examined to determine the cut-off, sufficient to rule out shedding of non-viable virus particles [12–24]. Since we did not have genotyping for confirmation of re-infection, we considered a negative result for SARS-CoV-2 RNA by any of the molecular tests after the confirmed first infection as indication of viral clearance.

In order to achieve the second objective, collective evidence from prospective cohort studies conducted across the globe were also taken into cognizance. Unique laboratory identification number was used to match the multiple tests of an individual. After obtaining verbal consent from eligible participants, a structured questionnaire was administered to collect data regarding symptomatology during the episodes and in the interim period, duration and health services utilisation. World Health Organization (WHO) ordinal scale for clinical improvement was used to assess the status of the participants during both the episodes. Archived data were used to record age, test dates, type of testing and cycle threshold (Ct) values. The ICMR-Central Ethics Committee on Human Research approved this study. Paired categorical variables were analysed using McNemar's test while continuous variables were analysed using paired *t* test (for normally distributed data) and Wilcoxon sign-rank test (for non-normally distributed data).

## Results

### Development of a working epidemiological definition

**Fig. 1.**
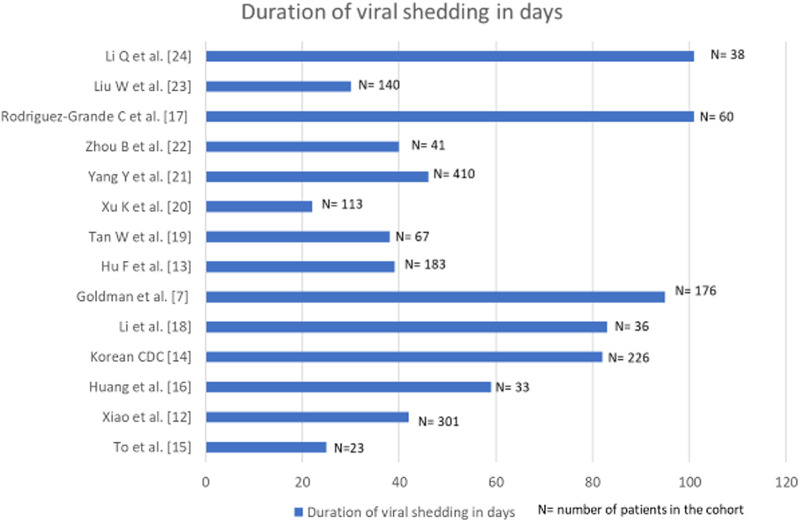
Bar diagram denoting the duration of viral shedding in days in cohorts where multiple longitudinal RT-PCRs for SARS-CoV-2 were done.

Prospective cohort studies from across the globe were collated and examined in depth. The findings from these investigations documenting the duration of viral shedding regardless of viability are graphically represented in Figure 1 [7, 12–24].

In a study from China, a cohort of 301 SARS-CoV-2 confirmed patients had multiple sequential viral RNA RT-PCR tests. The median time interval from symptom onset to the last positive RT-PCR result was 16 days (IQR: 10–23 days) [12]. Another cohort of 176 patients from the USA, with at least two positive viral RT-PCR documented a median duration between the first and the last positive RT-PCR as 12.1 (IQR: 6.4, 24.7) days. Duration between the first and the last positive RT-PCR was <59 days in 95% of the patients [7]. A Chinese cohort of 36 patients diagnosed before April 2020, documented viral RNA shedding for a median duration of 53.5 days (IQR 47.7- 60.5); the longest being 83 days [13]. The Korean Centre for Disease Control (CDC) reported that it took on an average 44.9 days (range: 8–82 days) from initial onset of symptoms to testing re-positive on RT-PCR after discharge. Viable virus could not be cultured from the samples collected in second episodes [14]. Relatively, smaller cohorts of 23 and 33 patients from Hong Kong and China recorded viral shedding for a maximum of 25 and 59 days, respectively [15, 16]. An Italian study reported the maximum days of viral shedding to be 101 in a cohort of 60 patients [17].

A meta-analysis of 43 investigations (including cohort studies and randomised-controlled trials) probed into the dynamics of viral shedding and reported the mean duration of detection of SARS-CoV-2 viral RNA from upper respiratory tract to be 17 days (95% CI: 15.5–18.6) with the maximum period of viral shedding being 83 days [25]. Persistent RT-PCR positivity for SARS-CoV-2 RNA has been reported up to 104 days in a solitary case report [26]. Taking all these available data into consideration, reinfection with SARS-CoV-2 in our study was defined as any individual who tested positive for SARS-CoV-2 on two separate occasions by either molecular tests or rapid antigen test at an interval of at least 102 days with one negative molecular test in between. Findings from cohort studies rather than isolated case reports formed the basis for such consideration.

### SARS-CoV-2 re-infection based on the derived case definition

Between 22nd January 2020 and 7th October 2020 (end date decided for the current investigation), 78 851 675 individuals were tested for COVID-19 in India. Considering the intervening period of 102 days, out of the 9 533 637 individuals tested before 30th June, 693 297 were eligible for re-infection study as they tested positive. Among those eligible, 91 592 (13.2%) individuals were tested at a gap of 102 or more days and therefore satisfied the criteria for being included in the investigation frame. Of these 91 592 individuals, who had another molecular test done in the interim gap of 102 days, were selected. We had records of 1300 (1.4%) such individuals. Fifty-eight of 1300 (4.5%) tested positive on two test occasions with 102 days interval and an interim negative test. Thirty-eight patients (38/58, 65.5%) could be contacted, provided consent and were included in this report. Twenty patients (20/58; 34.5%) could not participate as they had incorrect contact details in database (8), did not satisfy case definition (6), did not respond (5) and denied consent (1).

Most of the suspected cases of re-infection were male (29, 76.3%) and were in the 20–40 years age group (30, 78.9%). The mean (s.d.) age of study group was 34.4 (10.5) years (range: 21–67 year). Twelve of the 38 (31.6%) plausible re-infected cases were healthcare workers (HCWs). The median (IQR) duration between the two episodes of SARS-CoV-2 test positivity was 119 (108.75–144.25) days, ranging from 102 to 160 days. While majority of the participants remained asymptomatic (*n* = 27, 71.1.%) during the intervening period, there were eight participants who reported to be mildly symptomatic (fatigue) and three reported to be symptomatic (that included intermittent fever, cough or shortness of breath) during the intervening period.

Of the total suspected 38 cases of re-infection, a large proportion of the respondents were asymptomatic during the first episode (18, 47.4%) as compared to the second one (6, 15.8%). Of the 18 participants asymptomatic in first episode, 12 reported symptoms in the second episode. Amongst the 20 patients who were symptomatic at both episodes, six reported the second episode of infection as more severe than the first one, while another six reported the second episode as milder and the remaining reported both episodes of similar intensity. There were no significant differences between the two episodes with respect to duration of symptoms (Table 1). Of the 13 participants for whom paired Ct value for either of the confirmatory genes (RdRp/ORF) for both episodes were available, nine had a lower value in the second episode, of whom in six cases the first episode was asymptomatic and the second symptomatic. Paired archived RNA sample for both the episodes for whole genome sequencing were not available.

**Table 1.** Clinical and laboratory characteristics of study participants during the two episodes of SARS-CoV-2 infection

Clinical and laboratory characteristics	First episode of SARS-CoV-2 infection Number (%)	Second episode of SARS-CoV-2 infection Number (%)	*P* value
Asymptomatic	18 (47.4)	6 (15.8)	<0.001
Symptoms			
Cough	11 (28.9)	15 (39.5)	0.70
Fever	9 (23.7)	23 (60.5)	0.09
Sore throat	6 (15.8)	11 (28.9)	0.1
Other symptoms	12 (31.6)	14 (36.8)	Ref
Duration of symptoms in days, median (IQR), range	5 (2.75−7.75),	5 (3−7)	0.09
	2−14	2−20	
Type of test			
RT-PCR	37 (97.4)	31 (81.6)	0.07
RAT	1 (2.6)	7 (18.4)	
Ct values (screening E gene), mean (s.d.)	29.6 (6.9)	24.9 (5.6)	0.04
	*n* = 15	*n* = 17	
Ct values (RdRp gene), mean (s.d.)	26.4 (7.7)	24.3 (5.8)	0.9
	*n* = 12	*n* = 9	
Ct values (ORF1 gene), mean (s.d.)	32.4 (4.1)	26.2 (8.7)	0.31
	*n* = 12	*n* = 18	

Values are expressed as *n*(%) unless specified. RAT: rapid antigen test, RT-PCR: real-time reverse transcriptase polymerase chain reaction, Ct: cycle threshold.

Only one patient required mechanical ventilation in the second episode, none in the first episode.

## Discussion

This report shares the re-positivity of SARS-CoV-2 RNA testing conducted 102 or more days apart with a negative molecular test in the interim period. We found 38 such participants in our database who could be contacted for telephonic survey. More patients were symptomatic and Ct values were lower in the second episode than the first episode indicating higher viral load. Studies with confirmed cases of re-infection through genotyping have reported a similar phenomenon [7–9]. A brief synthesis of the 16 case reports of confirmed cases of SARS-CoV-2 reinfection published till date is presented in Supplementary Table 1. Most of these cases of confirmed re-infection except three occurred within 100 days of the first infection event. However, these reports did not provide information as to how long the shedding of viral RNA continued after the first instance of confirmed infection. Prolonged viral shedding has been associated with older age in studies which have prospectively followed patient cohorts [25]. The younger age of the participants of our report (mean: 34.4 year (s.d.:10.5); minimum: 21 year and maximum: 67 year) points against the 2nd positive molecular test being merely due to replication-incompetent viral shedding. Taking all the above in consideration, a gap of 102 days between two positive tests with a negative molecular test in the interim seemed appropriate for defining re-infection of SARS-CoV-2.

Some respondents in our study had a symptomatic second episode as opposed to the first one. The rate and duration of hospitalisation was not compared as during the initial phase of the pandemic in India all cases were being hospitalised for at least 14 days, irrespective of symptom severity. In our cohort of 38 patients, only six were asymptomatic during the second episode who were diagnosed during screening prior to travel or hospital admission for non-COVID-19 reasons.

Nearly 32% of our participants were HCWs. The previous six confirmed cases reported from India were all HCWs, as were the cases reported from Brazil [5, 6]. The finding could be interpreted as HCWs having continued high-risk occupational exposure and thus requiring surveillance more often. The present study is however limited by its descriptive nature to make a firmer inference regarding HCWs' vulnerability to SARS-CoV-2 re-infection.

This current study had some limitations, such as the absence of genome sequencing and the unknown antibody profile of the participants. The RNA samples are stored only for 6 weeks for quality assurance as deemed necessary by the national programme in India. Hence, next-generation sequencing could not be performed and may not be possible even in future at a population level, given the resource-intensive nature of such endeavour. The sensitivity of RT-PCR ranges from 73% to 97% with a chance of molecular test in the interim period being falsely negative. This could lead to prolonged viral shedding being picked up later as re-infection. However, using a time gap of 102 days would make such possibility highly unlikely as indicated by the worldwide observations. Another limitation was that paired Ct values of SARS-CoV2 RNA were available for only 13 participants.

Currently, there is no consensus regarding the working definition of re-infection, based only on epidemiological features; a resource-intensive method like whole genome sequencing being the only confirmation. It is not logistically feasible to store the samples of millions of positive cases for future sequencing to identify an important phenomenon like SARS-CoV-2 re-infection. Both CDC and European CDC suggested the use of genomic evidence for confirmation of re-infection. However, an epidemiological working definition will be more pragmatic and helpful to assess the magnitude of re-infection in most population and resource constrained settings.

While COVID-19 re-infection is still rarely reported, nonetheless, immunity should not be assumed and public health measures such as physical distancing, hand-hygiene and use of masks should be followed after recovery from first event of infection. Further well-designed cohort studies must be undertaken to understand the natural history of COVID-19 including its immunogenicity, susceptibility to re-infections, antibody-dependent enhancement and the severity of re-infections. It may also be suggested that the samples of HCWs may be stored for genomic analysis to study suspected COVID-19 reinfections, particularly in resource-limited settings as chances of them encountering such events are higher due to potential high-risk occupational exposure.

## Data Availability

All materials needed to replicate the findings of the article are available as Supplementary Materials. Readers can also contact the authors for the same.

## Supplementary material

For supplementary material accompanying this paper visit http://dx.doi.org/10.1017/S0950268821000662. click here to view supplementary material
