# Supplementary material for: SARS-CoV-2 re-infection: development of an epidemiological definition from India
Source: Epidemiol Infect. 2021 Mar 26;149:e82. doi: 10.1017/S0950268821000662 (PMC8027559; doi:10.1017/S0950268821000662)
Supplement: Supplementary file 1 [file S0950268821000662sup001.docx]

Supplemental Data

Supplemental Table 1: List of published reports of confirmed re-infection cases (by Next Generation Genomic Sequencing) of COVID-19 across the world

| Country (reference) | Number of cases, age, general health status | Interval period | Clinical description of 2 episodes |
| --- | --- | --- | --- |
| Hong Kong^1^ | 1, 33Y old immune-competent male | 148 days | 1^st^ episode: Ct:30.5  2^nd^ episode: Ct: 32 |
| USA (Nevada)^2^ | 1, 25Y old immune-competent male | 48 days | 1^st^ episode: Ct:35.2  2^nd^ episode: Ct: 35.3 |
| Belgium^3^ | 1, 51Y old female on daily inhaled steroids | 93 days | 1^st^ episode: Ct:25.6  2^nd^ episode: Ct: 32.6 |
| Ecuador^4^ | 1, 46Y old immune-competent male | 63 days | 1^st^ episode: Ct:36.85 |
| India^5^ | 2, 25Y and 28 Y old immune-competent males | 108 days  111 days | First case  1^st^ episode: Ct:36  2^nd^ episode: Ct: 16.6  Second case  1^st^ episode: Ct:28.16  2^nd^ episode: Ct: 16.92 |
| India^6^ | 4, 27 Y, 31 Y and 27 Y old male and 24 Y female health care workers | 66 days  65 days  19 days  55 days | First case  1^st^ episode: Ct:32  2^nd^ episode: Ct: 25  Second case  1^st^ episode: Ct: 33  2^nd^ episode: Ct: 36  Third case  1^st^ episode: Ct:36  2^nd^ episode: Ct: 21  Fourth case  1^st^ episode: Ct:32  2^nd^ episode: Ct:17 |
| USA^7^ | 1, 60-69 Y, male | 140 days | 1^st^ episode: Ct:26.5  2^nd^ episode: Ct: 39.6 |
| Qatar^8^ | 4, 45-49 Y female, two 25-29 Y males, 40-44 Y male, | 88 days  46 days  71 days  55 days | First case  1^st^ episode: Ct:36  2^nd^ episode: Ct: 25  Second case  1^st^ episode: Ct: 36  2^nd^ episode: Ct: 28  Third case  1^st^ episode: Ct: 17  2^nd^ episode: Ct: 29  Fourth case  1^st^ episode: Ct:30  2^nd^ episode: Ct:32 |
| France^9^ | 1, 70 Y old immune competent male | 105 days | 1^st^ episode: Ct:27  2^nd^ episode: Ct: 18 |

Ct: Cycle Threshold; Y: years
